# Evaluating the impact of a maternal health voucher programme on service use before and after the introduction of free maternity services in Kenya: a quasi-experimental study

**DOI:** 10.1136/bmjgh-2018-000726

**Published:** 2018-05-02

**Authors:** Mardieh L Dennis, Timothy Abuya, Oona Maeve Renee Campbell, Lenka Benova, Angela Baschieri, Matteo Quartagno, Benjamin Bellows

**Affiliations:** 1 Faculty of Epidemiology and Population Health, London School of Hygiene and Tropical Medicine, London, UK; 2 Population Council Kenya, Nairobi, Kenya; 3 Population Council Zambia, Lusaka, Zambia

**Keywords:** health systems, maternal health, intervention study, community-based survey

## Abstract

**Introduction:**

From 2006 to 2016, the Government of Kenya implemented a reproductive health voucher programme in select counties, providing poor women subsidised access to public and private sector care. In June 2013, the government introduced a policy calling for free maternity services to be provided in all public facilities. The concurrent implementation of these interventions presents an opportunity to provide new insights into how users adapt to a changing health financing and service provision landscape.

**Methods:**

We used data from three cross-sectional surveys to assess changes over time in use of 4+ antenatal care visits, facility delivery, postnatal care and maternal healthcare across the continuum among a sample of predominantly poor women in six counties. We conducted a difference-in-differences analysis to estimate the impact of the voucher programme on these outcomes, and whether programme impact changed after free maternity services were introduced.

**Results:**

Between the preintervention/roll-out phase and full implementation, the voucher programme was associated with a 5.5% greater absolute increase in use of facility delivery and substantial increases in use of the private sector for all services. After free maternity services were introduced, the voucher programme was associated with a 5.7% higher absolute increase in use of the recommended package of maternal health services; however, disparities in access to facility births between voucher and comparison counties declined. Increased use of private sector services by women in voucher counties accounts for their greater access to care across the continuum.

**Conclusions:**

Our findings show that the voucher programme is associated with a modest increase in women’s use of the full continuum of maternal health services at the recommended timings after free maternity services were introduced. The greater use of private sector services in voucher counties also suggests that there is need to expand women’s access to acceptable and affordable providers.

**Key questions**

**What is already known?**

- Previous research examining the short-term effects of this reproductive health voucher programme suggests that the programme is associated with increased use of facility delivery and private sector maternal health services.
- Studies on the free maternity services policy in Kenya also suggest that the policy has increased use of facility delivery.

**What are the new findings?**

- We found that while disparities in access to facility birth decreased between voucher and comparison counties after the introduction of free maternity services, births in voucher counties were more likely to have received a full package of 4+ antenatal care, facility delivery and postnatal care at the recommended timings.
- Greater use of private sector providers in voucher counties accounts for the differences in levels of access to the full continuum of maternal health services both before and after the introduction of free maternity services.

**What do the new findings imply?**

- The findings suggest that even when services are free in the public sector, when given a choice of affordable providers, a substantial proportion of poor women choose to complement public services with care in the private sector or exclusively seek care in the private sector.
- To ensure further reductions in maternal mortality, policy-makers must better understand when and why women choose to seek private sector care and consider how to engage high-quality private sector providers to equitably reach women of all socioeconomic groups who would otherwise not access care along every point in the maternal healthcare continuum in a timely manner.

## Introduction

Although maternal mortality has decreased substantially around the world over the past three decades, additional reductions are a top priority for the global development agenda.^1^ In 2015, an estimated 303 000 women died from complications related to childbirth, largely from preventable causes.^2 3^ The burden of poor maternal health is particularly acute in sub-Saharan Africa, where the maternal mortality ratio (MMR) of 546 deaths per 100 000 live births is 2.5 times greater than the global MMR and 46 times greater than that of high-income countries.^3^ Despite the consensus on effective interventions for reducing the risks associated with pregnancy and childbirth, many women in low-income and middle-income countries (LMICs) do not access high-quality maternal health services due to a number of barriers, including limited availability, lack of transportation and high cost of care.^4 5^

In Kenya, the MMR declined from 590 maternal deaths per 100 000 live births in 1998 to 362 in 2014.^6 7^ Since independence in 1963, the Kenyan government has implemented a series of user fee introductions, reductions and removals in an effort to strike a balance between ensuring adequate cost recovery for health facilities and affordable, universal access to essential services, including maternal healthcare, for individuals.^8–12^ Nevertheless, according to the 2014 Kenya Demographic and Health Survey, nearly two in every five Kenyan women still reported giving birth outside of a health facility or without the supervision of a skilled birth attendant. The survey also found pronounced inequity in access to maternal health services in Kenya, with 70% of women in the poorest wealth quintile delivering under these suboptimal conditions compared with only 7% of women in the highest quintile.^6^

Given persistent disparities, the Government of Kenya has piloted alternative health financing approaches to further reduce financial barriers and ensure universal access to care.^13^ One such strategy, the reproductive health voucher programme, aimed to make high-quality maternal health, family planning and gender-based violence services more available and affordable for poor women.^14 15^ On the demand side, this programme sought to reduce women’s expenditures on maternal health services by selling highly subsidised safe motherhood vouchers that covered care across the maternal health continuum, including four antenatal care (ANC) visits, facility delivery (vaginal or caesarean) and postnatal care (PNC). These vouchers were sold for KES200 (equivalent to 2006 USD$2.70/2016 USD$1.94) and were intended to be specifically targeted to poor women, as determined by a poverty grading assessment administered to each potential user. On the supply side, the voucher programme sought to expand provider choice and improve quality of care by enrolling both public and private sector lower levels and referral facilities into the programme. Facilities that met certain minimum standards could be accredited for participation in the programme and were reimbursed at standard, prenegotiated rates for each voucher service provided. Additionally, periodic quality assurance assessments were conducted, and facilities that failed to uphold the minimum standards risked losing their accreditation. The voucher programme was implemented in phases from 2006 to 2016 and managed by PriceWaterhouseCoopers on behalf of the Kenyan government with support from the German Development Bank (KfW). In the first phase, from 2006 to 2009, the voucher intervention was piloted in four counties (Kiambu, Kisumu, Kitui and Nairobi). Following the pilot, the programme was expanded to an additional county (Kilifi) as well as to additional facilities in the pilot counties, and implementation continued until late 2016.^14^

During the final implementation phase of the voucher programme, on 1 June 2013, the Government of Kenya announced a major maternal health financing policy change: maternity services were to be provided for free in all public health facilities across the country with immediate effect. Facilities were to provide free maternal healthcare to all women and receive a standard reimbursement from the government for services provided. Thus, for over 3 years between 2013 and 2016, the voucher and free maternity services programmes operated concurrently.

The unexpected and concurrent implementation of these two interventions is reflective of the challenges of real-world programme evaluations and presents a unique opportunity to provide new insights into how health systems and users adapt to a changing landscape of health financing and service provision. Previous studies have explored the shorter-term effects of the Kenya voucher programme on maternal health service utilisation, out-of-pocket expenditures and quality of care.^16–19^ Building on this evidence base, this study aims to examine the longer-term impact of the voucher programme on maternal health service utilisation and to assess whether any observed effects of the voucher programme persisted after free maternity services were introduced in 2013.

## Methods

### Study design and setting

A quasi-experimental study was conducted with repeated cross-sectional surveys administered in May 2010–July 2011, July–October 2012 and July–August 2016. Data were collected in four intervention counties (Kiambu, Kilifi, Kisumu and Kitui) and three comparison counties (Makueni, Nyandarua and Uasin Gishu) selected to match the geographical, population and health facility characteristics (type of facility and ownership) of the intervention counties. To facilitate comparisons over time, one intervention county (Kilifi) was excluded from this analysis, as it was not surveyed in 2016. We included a map of the study counties in online supplementary appendix 1).

10.1136/bmjgh-2018-000726.supp1 Supplementary data

The study used a multistage sampling design. In the first stage, a random sample of 14 sublocations were selected within each intervention county from those located within a 5 km radius of a facility accredited in the voucher programme. In comparison counties, 14 sublocations were selected among those within a 5 km radius of a facility that were comparable to the intervention facilities in terms of facility type and ownership. This was done to ensure that all surveyed women had similar physical access to the maternal health services offered under the voucher programme. At the second sampling stage, three villages were randomly selected within each sublocation. Given that the voucher programme intended to target poor women, the poorest households in each village were identified by local administrators and purposively selected for inclusion in the study. Within each household, women aged 15 to 49 years with at least one birth in the past 12 months or pregnant at the time of the interview were targeted for participation. In households with more than one woman meeting the target characteristics, the youngest woman was selected into the study. Additional details of the study protocol and sampling methods have been described previously.^16–18 20^

Face-to-face interviews were conducted during each survey round using a tablet-based structured questionnaire covering a range of topics including women’s sociodemographic characteristics, reproductive history and maternal health service utilisation. Each participant provided written informed consent to participate in the study.

### Study outcomes

Table 1 defines the 10 indicators of maternal health service utilisation and sector of care examined in this study. In addition to examining use of individual services in each period, we also looked at the proportion of women receiving a complete package of all three services across the maternal health service continuum (complete care). We also estimated the proportion receiving complete care at the recommended timings, with the first ANC visit occurring during the first trimester and the PNC check occurring within 48 hours of delivery (recommended care).

**Table 1:** Indicator definitions

Service utilization	
4+ ANC visits	Births for which a woman attended four or more ANC visits were categorised as having received 4+ ANC visits. Births with missing information on the number of ANC visits were considered to have not received 4+ ANC visits.
Facility delivery	All births that occurred in a health facility, regardless of birth attendant or sector of care, were categorised as facility deliveries. Births with missing information on delivery location were considered to have not occurred in a health facility.
Postnatal care	Births after which a woman reported a health worker checking on her health were categorised as having received PNC. Births with missing information on receipt of a PNC check were considered to have not received PNC.
Complete care	Births that received: (a) 4+ ANC visits and (b) Facility delivery and (c) Postnatal care for mother
Recommended care	Births that received: (a) 4+ ANC visits, with the first visit occurring in the first trimester and (b) Facility delivery and (c) Postnatal care for mother within 48 hours of delivery

Sector of care	
Public sector	Births that received a given maternal health service in a government-owned facility were categorised as having received care in the public sector. Births that received care in a facility owned by a non-government actor, at home or with missing information (<1%) on sector of care were categorised as not having received care in the public sector.
Private sector	Births that received a given maternal health service in a private for-profit, non-profit or faith-based facility were categorised as having received care in the private sector. Births that received care in a government-owned facility, at home or with missing information (<1%) on sector of care were categorised as not having received care in the private sector.
All public	Births that received ANC, delivery and PNC services all in the public sector among users of recommended care. This category also includes a small number (n=4) of public facility births that received home-based ANC and/or PNC.
All private	Births that received ANC, delivery and PNC services all in the public sector among users of recommended care.
Both public and private	Births that received ANC, delivery and PNC services from both public and private sector sources among users of complete or recommended care.

ANC, antenatal care; PNC, postnatal care.

### Statistical analysis

Respondents were asked to report on all of their births within the 5 years prior to the survey; data from the three cross-sectional surveys were pooled and reshaped to allow us to perform analyses on all reported births. We categorised these births into three periods according to when they occurred. Period 1 (May 2005–December 2009) refers to the pre-intervention and roll-out phase of the programme. Period 2 (January 2010–May 2013) refers to the post roll-out phase, when the programme was implemented at full intensity. Lastly, Period 3 (June 2013–August 2016) refers to the period when both the voucher programme and the free maternity services policy for all government facilities were being implemented simultaneously.

For the data collected in 2016, a glitch in the survey programming resulted in 23% of women who reported giving birth at least once in their lifetime having a missing response to the question, ‘During the last 5 years, how many children have you given birth to?’ This question was missing for less than 1% of respondents in both the 2010 and 2012 surveys. Based on the skip pattern of the instrument, only women who reported giving birth to one or more child in the past 5 years were asked subsequent questions about the key outcomes of this study related to maternal health service utilisation for each child born within the period. Women who reported zero births or had missing information on their number of births in the past 5 years were not asked these questions; we are therefore missing outcome data for births that occurred within the past 5 years to women with missing information for the aforementioned question.

We conducted analyses to explore for any evidence of systematic biases in our estimates relating to the pattern of missing data in the question about the number of live births 5 years prior to the survey (online supplementary appendix 1). We found that after controlling for all relevant sociodemographic characteristics, both marital status and county had strong effects on the odds of having missing data. The observed effect of county is due to the fact that the data manager identified the glitch during the course of fieldwork and corrected it; the proportion of missing data therefore declined after the instrument was updated (Table A2.1). The mechanism behind the effect of marital status is unclear and may be due to chance. These findings suggest that the data are not missing completely at random and might either be missing at random (MAR) conditional on both county and marital status or missing not at random. However, because we know that the missing data mechanism was due to a software issue that is unrelated to the underlying values of the our outcomes of interest, we have assumed the data to be MAR and have conducted a complete case analysis controlling for both county and marital status.^21 22^ Less than 1% of responses were missing for all other variables across all three surveys.

10.1136/bmjgh-2018-000726.supp2 Supplementary data

We performed Wald tests to assess cross-sectional differences in background characteristics between all surveyed women in voucher and comparison counties for each period. We used logistic regression models, adjusted by background characteristics, to estimate cross-sectional differences in women’s maternal health service utilisation for births that occurred in voucher and comparison counties. Our analysis of women’s background characteristics used a logistic regression models adjusted for multistage clustering at the sublocation and village levels. Outcomes related to service utilisation additionally accounted for clustering at the mother level, as some women reported more than one live birth within the 5 years prior to the survey.

We used a difference-in-differences approach with mixed-effects linear regression models to approximate the impact of the voucher programme and introduction of free maternity services on maternal health service utilisation and sector of care with random effects included for county sublocation, village and mother. To assess the impact of the voucher programme, we estimated differences in the change over time in outcomes between births that occurred in voucher and comparison counties before (Period 1) and after (Period 2) the voucher programme was fully implemented. We further assessed whether any benefits of the voucher programme persisted after free maternity services were introduced by estimating the difference in the change in outcomes between births in voucher and comparison counties before (Period 2) and after (Period 3) user fees were removed.

We present these voucher programme impact results controlled for key potential confounders, including location (urban/rural), wealth quintile, year of childbirth, insurance enrolment, mother’s parity, education, marital status and employment status.

We used STATA IC V.15.1 (StataCorp LLC) to conduct this analysis.

## Results

A total of 7136 births from 5323 women were included. Across voucher and comparison groups and over time, the births in our sample were predominantly to women living in rural areas who were married, multiparous, educated to the primary school level or below, unemployed or informally employed and uninsured (table 2). Within each period, the women sampled from the voucher and comparison counties were similar with regard to many background characteristics. However, in Period 1, women from voucher counties were less likely to have completed secondary education or higher, and Periods 1 and 3, women from voucher counties were more likely to be younger than women from comparison counties. In Period 2, women from voucher counties were more likely to be unmarried and unemployed. Additionally, in Periods 1 and 2, women from voucher counties were less likely to have health insurance coverage.

**Table 2:** Women’s background characteristics by study period

	Period 1 (Pre-voucher/roll-out period) n=1888			Period 2 (Full voucher implementation) n=2198			Period 3 (Free maternity services introduced) n=1237		
	Comparison counties	Voucher counties	P values	Comparison counties	Voucher counties	P values	Comparison counties	Voucher counties	P values
Age group (years) (%)			P=0.002			P=0.079			P=0.018
15–24	23.1	32.3		32.5	38.8		32.8	39.5	
25–34	50.6	48.9		49.3	46.2		50.1	45.1	
35+	26.3	18.9		18.3	15.0		17.1	15.6	
Educational attainment (%)			P=0.021			P=0.351			P=0.382
Below primary	26.2	32.2		28.1	32.6		24.3	27.6	
Completed primary	58.1	55.3		53.6	51.2		51.5	47.4	
Completed secondary/higher	19.7	12.5		18.3	16.2		24.2	25.0	
Wealth quintile (%)			P=0.089			P=0.786			P=0.505
Poorest	18.1	20.3		21.1	20.1		17.7	22.3	
Poorer	19.6	21.7		22.4	20.6		22.8	20.0	
Middle	22.2	20.9		19.0	18.8		19.1	19.2	
Richer	19.8	18.4		18.1	21.4		22.1	19.7	
Richest	20.3	18.7		19.4	19.1		18.2	18.8	
Residence			P=0.478			P=0.365			P=0.587
Rural	87.5	82.4		87.4	80.1		90.2	85.9	
Urban	12.5	17.6		12.6	19.9		9.8	14.1	
Current marital status (%)			P=0.265			P=0.014			P=0.957
Unmarried	16.7	19.1		16.1	20.8		22.5	22.3	
Married/cohabiting	83.3	80.9		83.9	79.2		77.5	77.7	
Woman’s employment (%)			P=0.453			P=0.022			P=0.140
Unemployed	34.6	39.2		40.4	50.4		45.4	51.3	
Informally employed	43.6	41.1		48.0	39.1		48.1	39.8	
Formally employed	21.8	19.7		11.5	10.6		6.4	8.8	
Parity (%)			P=0.451			P=0.484			P=0.978
1 child	17.7	20.5		21.1	23.6		27.9	27.3	
2–3 children	44.2	43.4		43.6	43.0		44.6	45.0	
≥4 children	38.1	36.2		35.3	33.4		27.5	27.8	
Health insurance enrolment (%)			P<0.001			P=0.032			P=0.283
Uninsured	86.5	93.4		86.3	90.8		79.7	82.8	
Insured	13.5	6.6		13.7	9.2		20.3	17.2	
Total no of women	871	1017		1066	1132		592	645	

## Service utilisation

**Figure 1:**
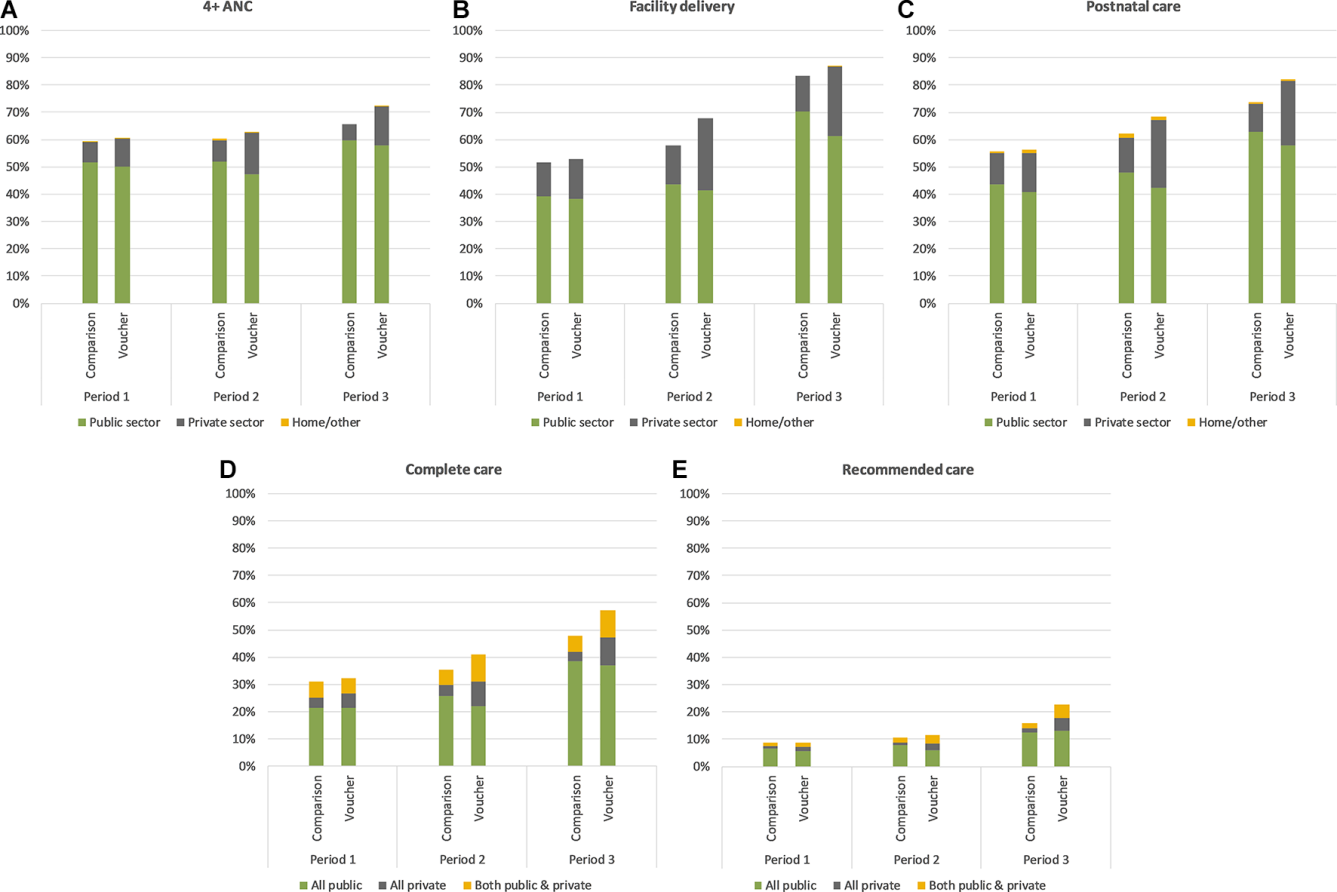
Use of maternal health services over time. ANC, antenatal care.

Women in both voucher and comparison counties reported receiving 4+ ANC visits for 59.4% to 62.7% of the births that occurred during Periods 1 and 2 (figure 1A); this increased moderately after free maternity services were introduced (Period 3). We estimated the odds ratio (OR) of attending 4+ ANC visits adjusted for differences in key sociodemographic background characteristics and found that while use of 4+ ANC was similar in voucher and comparison counties in Periods 1 and 2, a greater proportion of births in voucher counties received 4+ ANC visits in Period 3 (OR 1.46, p=0.006) (table 3).

**Table 3:** Adjusted cross-sectional comparison of service utilisation and source of care in voucher versus comparison counties

	Period 1		Period 2		Period 3	
	Adjusted OR* (95% CI)	P values	Adjusted OR* (95% CI)	P values	Adjusted OR* (95% CI)	P values
Service utilisation						
4+ ANC visits	1.12 (0.94 to 1.34)	0.201	1.18 (0.99 to 1.40)	0.072	1.46 (1.11 to 1.90)	0.006
Facility delivery	1.18 (0.85 to 1.64)	0.315	1.65 (1.14 to 2.37)	0.008	1.47 (0.91 to 2.39)	0.115
PNC	1.13 (0.89 to 1.46)	0.308	1.37 (1.01 to 1.86)	0.043	1.73 (1.25 to 2.40)	0.001
Complete care	1.20 (0.95 to 1.51)	0.130	1.34 (1.02 to 1.75)	0.037	1.58 (1.20 to 2.10)	0.002
Recommended care	1.02 (0.75 to 1.41)	0.871	1.07 (0.79 to 1.44)	0.674	1.68 (1.23 to 2.31)	0.001
Private sector market share						
ANC†	1.46 (0.86 to 2.48)	0.158	2.11 (1.27 to 3.49)	0.004	2.71 (1.38 to 5.31)	0.004
Facility delivery	1.32 (0.84 to 2.07)	0.220	2.02 (1.33 to 3.07)	0.001	2.26 (1.36 to 3.73)	0.002
PNC	1.44 (0.92 to 2.28)	0.110	2.44 (1.55 to 3.84)	<0.001	2.59 (1.47 to 4.54)	0.001
Complete care‡	1.33 (0.89 to 2.00)	0.167	2.45 (1.58 to 3.78)	<0.001	2.51 (1.50 to 4.20)	0.001
Recommended care‡	1.70 (0.88 to 3.27)	0.112	2.59 (1.45 to 4.61)	0.001	3.04 (1.43 to 6.46)	0.004

*Logistic regression model adjusted for woman’s age at birth, education, wealth, residence, marital status, employment, parity and multistage sampling at the county sublocation, village and mother levels.

†Among users of 4+ ANC visits.

‡Proportion of users who received care from the private sector for at least one service in the 4+ ANC, delivery care and PNC continuum.

ANC, antenatal care; PNC, postnatal care.

Delivery in health facilities increased from approximately half of all births in Period 1 to 83.2% (comparison counties) and 86.7% (voucher counties) of births in Period 3 (figure 1B). Although there was no difference in utilisation of facility delivery between voucher and comparison counties in Periods 1 and 3, a greater proportion of births in Period 2 were delivered in health facilities in voucher counties than in comparison counties (OR 1.65, p=0.008) (table 3).

Use of postnatal care services for the mother increased steadily from nearly 60% of all births in Period 1 to 73.9% and 82.1% of births in comparison and voucher counties in Period 3, respectively (figure 1C). In Period 3, births in voucher counties were more likely to have received PNC than those in comparison counties (OR 1.73, p=0.001) (table 3).

In both voucher and comparison counties and across time, the proportion of women who reported receiving either 4+ ANC visits, facility delivery or PNC for their births individually substantially exceeded the proportion who received complete care, defined as all three services across the maternal healthcare continuum for a single birth (figure 1D). For instance, while over 80% of births reported in Period 3 were delivered in health facility, only 47.7% of births in comparison counties and 57.3% of births in voucher counties received complete care during that period. Further, an even smaller proportion of births received care both across the continuum and at the recommended timings. In Period 1, fewer than 10% of births in both intervention groups received recommended care (figure 1E). Use of recommended care increased over time so that by Period 3, a greater proportion of births in voucher counties received recommended care than in comparison counties (OR 1.68, p=0.001) (table 3).

## Sector of care

The public sector was consistently the predominant provider of maternal health services for our sample; in each period, less than 40% of ANC, facility delivery and PNC users reported receiving care from the private sector (figure 1A–C). However, in all periods, the proportion of complete and recommended care users who sought care from the private sector for at least one service across the continuum was higher than the private sector market share for each of the three services individually (figure 1A–E).

In Period 1, prior to the full implementation of the voucher programme, there was no difference in use of the private sector for maternal health services individually or as a package between voucher and comparison counties in Period 1. The private sector market share increased substantially between Periods 1 and 2 in voucher counties, such that the proportion of all types maternal healthcare received from the private sector was significantly higher in voucher counties than in comparison counties in Period 2. Between Periods 2 and 3, private market share for all services declined in both voucher and comparison counties; however, use of the private sector remained significantly higher in voucher counties (table 3).

## Impact of voucher programme and free maternity services policy

We found no effect of the voucher programme or free maternity services policy on the use of 4+ ANC visits or receipt of PNC checks (table 4). The increase in the proportion of births that were delivered in a health facility between the pre-intervention/roll-out phase (Period 1) and the post roll-out phase (Period 2) was 5.5 percentage points greater (p=0.011) in voucher counties than in comparison counties. However, the results from Period 3 suggest that the free maternity services policy decreased the disparities in access to facility births between voucher and comparison counties, and births in comparison counties may have experienced a greater increase in facility deliveries than those in voucher counties once the free maternity services policy was introduced. As a result, we found no difference in the utilisation of facility delivery care between voucher and comparison counties in Period 3 (table 3).

**Table 4:** Impact of voucher programme and free maternity policy on service utilisation and source of care

	Period 1–Period 2		Period 2–Period 3	
	D-in-D estimator* (95% CI)	P values	D-in-D estimator* (95% CI)	P values
Service utilisation				
4+ ANC visits	0.012 (−0.035 to 0.059)	P=0.619	0.047 (−0.012 to 0.105)	P=0.119
Facility delivery	0.055 (0.013 to 0.098)	P=0.011	−0.049 (−0.102 to 0.003)	P=0.064
PNC	0.038 (−0.005 to 0.081)	P=0.083	0.009 (−0.045 to 0.063)	P=0.733
Complete care	0.021 (−0.024 to 0.066)	P=0.366	0.045 (−0.011 to 0.101)	P=0.117
Recommended care	0.000 (−0.031 to 0.031)	P=0.999	0.057 (0.018 to 0.096)	P=0.004
Private sector market share				
ANC†	0.075 (0.043 to 0.106)	P<0.001	0.025 (−0.015 to 0.066)	P=0.218
Facility delivery	0.105 (0.049 to 0.160)	P<0.001	0.000 (−0.059 to 0.059)	P=1.000
PNC	0.110 (0.058 to 0.162)	P<0.001	−0.001 (−0.067 to 0.048)	P=0.744
Complete care‡	0.147 (0.073 to 0.222)	P<0.001	−0.008 (−0.086 to 0.070)	P=0.842
Recommended care‡	0.181 (0.045 to 0.317)	P=0.009	−0.030 (−0.160 to 0.100)	P=0.652

*Mixed-effects linear regression model adjusted for child’s birth year, woman’s age at birth, education, wealth, residence, marital status, employment, parity and random effects at the county sublocation, village and mother levels.

†Among users of 4+ ANC visits.

‡Proportion of users who received care from the private sector for at least one service in the 4+ ANC, delivery care and PNC continuum.

ANC, antenatal care; PNC, postnatal care.

We did not observe any differences in the improvements over time in access to complete care between births that occurred in voucher and comparison counties. Although access to the recommended package of ANC, delivery and PNC services at the correct timings was low in all study counties, we observed a 5.7 percentage point greater improvement (p=0.004) in use of recommended care among births that occurred in voucher counties between Periods 2 and 3 (table 4).

Between Periods 1 and 2, we observed 7.5%–11.0% greater absolute increases (p<0.001) in the proportion of ANC, facility delivery and PNC users seeking care in the private sector in voucher counties than in comparison counties (table 4). Among users of complete and recommended care, increases in the use of private sector services at some point along the maternal healthcare continuum were 14.7 (p<0.001) and 18.1 (p=0.009) percentage points higher in voucher counties than in comparison counties between Periods 1 and 2, respectively. Use of private sector facilities appears to have decreased for all services types between Periods 2 and 3, and there was no evidence of differences in the change in use of private sector care between voucher and comparison counties after the introduction of free maternity services.

## Discussion

These results suggest that between the pre-intervention/roll-out and full implementation phases, the Kenya voucher programme modestly increased use of facility deliveries and stimulated a shift towards greater use of private sector providers for ANC, delivery and PNC services among a sample of predominantly poor women. However, after free maternity services were introduced, use of facility-based deliveries in comparison counties improved to levels similar to those observed in voucher counties, and there was greater use of public sector facilities for maternal health services across all counties. Although use of private sector services decreased universally after free care was introduced in government facilities, women in voucher counties continued to use the private sector at much higher levels than women in comparison counties after the policy change. Still, across all counties, periods and service types, the public sector remained the majority provider of maternal healthcare.

We did not find any positive impact of the voucher programme on access to 4+ ANC, facility delivery or PNC services individually after free maternity services were introduced. While we similarly did not find any impact on the collective use of all three services across the continuum after the policy change, we found a greater increase in use of the recommended care package of all three maternal health services at the correct timings among births in voucher counties. Qualitative evidence from Kenya suggests that the free maternity services programme overburdened public health facilities, resulting in reduced health worker motivation and quality of care.^23–25^ Our findings suggest that differences in use of recommended care may be partially explained by the greater ability of women in voucher counties to complement public sector services with care in the private sector or exclusively seek care in the private sector, after free maternity services were introduced. However, given the difference in the observed trends in use of complete compared with recommended care, further research is needed to better understand how factors such as women’s perceptions of quality of care and ability to pay may have encouraged more timely care seeking across the maternal health continuum.

Our finding that the voucher programme moderately increased the proportion of births that occurred in health facilities between the preintervention/roll-out and full implementation periods is consistent with previously reported results from evaluations of maternal health voucher programmes from Kenya and other LMICs.^16 17 26–28^ While other LMIC studies have inferred similar increases in access to 3+ or 4+ ANC and PNC services due to voucher programmes, we did not find such an effect.^28–34^ These results are also consistent with previous studies that have shown that offering affordable vouchers that can be redeemed in private facilities leads to greater use of private sector maternal health services.^16 17 29^ To our knowledge, this is the first study from an LMIC to examine the impact of the voucher programme on utilisation of care across the ANC, delivery and PNC service continuum.

This study has some key strengths that help to extend the body of knowledge generated by previous research on health voucher programmes in LMICs. First, most studies on voucher programmes to date have examined the immediate or shorter-term impact of the intervention on service utilisation.^28^ Ours is unique in that it looks at the mid-term to longer-term effects of the intervention and also examines how the voucher programme performs against an alternative health financing strategy. Additionally, much of prior research on the effect of voucher programmes on ANC, facility delivery and PNC utilisation from Kenya and other LMICs has relied on with-and-without and before-and-after study designs.^16 17 26–28^ Both of these analytical approaches rely on key assumptions for causal inference that are often invalid in observational studies—namely, that there are no underlying differences between the intervention and comparison groups related to the outcomes of interest and that without the intervention, there would be no differences in the outcome among study participants observed before and after implementation.^35^ This study overcomes some of the biases introduced by these assumptions by using a difference-in-differences approach that compares the difference in the change in maternal health service utilisation between treatment and comparison groups.

Despite these strengths, our study also has some important limitations. For instance, three aspects of the sampling approach were non-random. First, only villages located within a 5 km radius of a voucher-accredited or similar health facility were included in the sample; we are therefore unable to assess the impact of the programme in more remote areas. Thus, we may be overestimating the population-level effects of the programme by only evaluating impact among communities within close proximity of maternal health services. This, along with the fact that our survey was implemented more than 3 years after the policy change, might help explain why more than 80% of women in both voucher and comparison counties reported giving birth in a health facility after free maternity services were introduced, while the national estimate from the 2014 Kenya Demographic and Health Survey is only 61%. Second, within each village, the research team purposively sampled the poorest parts of the community in order to ensure that the interviewers surveyed an adequate number of women meeting the poverty criteria for participation in the voucher programme. As a result, we are unable to accurately assess the impact of the programme on equity in access to care, given that the sample predominantly includes women of similar socioeconomic status who were selected based on community leaders’ subjective understanding of their poverty status. Lastly, within each household, the youngest woman was selected if more than one eligible woman was present, which may also have introduced some age-related biases into our analyses.

Another limitation of this study is that we assessed the impact of the voucher programme at the community level, which is greatly affected by the penetration of the intervention. A previous study on the Kenya voucher programme found that 15.4% of women in voucher counties reported using a safe motherhood voucher during the 2010/11 survey and 43.9% reported using the voucher in the 2012 survey.^18^ This approach therefore likely underestimates the direct effects of the voucher programme on voucher users. A fundamental assumption of the difference-in-differences approach is that we would expect to observe equal trends over time in key outcomes between the treatment and comparison groups were it not for the intervention.^35^ However, due to the observational nature of this study, it is possible that this assumption may have been violated by the presence of other maternal health-related interventions or differential implementation of relevant policies in the study counties. For instance, the Kenyan government was decentralised in 2013, and since then, each county has semi-autonomously managed its own health system. Many counties have experienced challenges with this transfer of power that have contributed to reduced staff motivation, health worker strikes and lower quality of care; all of which may have affected the observed effects in our study.^36–38^

In terms of data quality, a glitch in the programming of the tablet-based survey instrument led to a significant amount of missing data for the 2016 survey. This resulted in a reduced sample size and loss of statistical power in Period 3, which may have affected our ability to detect differences by intervention group in women’s background characteristics and use of services in Period 3 (table 2 and 3) and in changes over time in maternal health service use between Periods 2 and 3 (table 4). Although the missing data may also introduce concerns about bias, we address this by accounting for clustering within counties and including marital status as a covariate in our models. Complete case analysis is valid when the outcome of the model is not included in the missing data mechanism; this is the case in our study, as the data are MAR when conditioned on the relevant covariates.^22 39^ Multiple imputation techniques have been gaining popularity over the last years for recovering information from incomplete records, particularly covariates; however, in our setting, data are missing only in the outcome, and therefore multiple imputation would not be useful.^39^

Despite these limitations, our study has important implications for health policy and financing in Kenya. The particularly important role that private sector services played in helping poor women to access the recommended care package in voucher counties before and after the introduction of the free maternity services policy suggests that the private sector can help to expand timely access to the full continuum of care, even when services are provided for free in the public sector. However, additional research should be conducted to clarify the underlying mechanisms influencing when and where women seek maternal health services under the free maternity policy, as decreased quality of care in the public sector may compel women who should benefit from free maternity services to seek care from facilities where they will incur out-of-pocket expenditures.

A large proportion of the health infrastructure in Kenya is operated by non-government for-profit, non-profit and faith-based actors, and it is estimated that more than 40% of all health services are provided by the private sector.^8 40^ Although these providers are often thought to serve the interests of higher income populations, our study demonstrates clear demand for private sector services in lower income, remote areas. These findings therefore support the Kenyan government’s recent decision to expand the free maternity services policy through the Linda Mama programme. Through this programme, the Kenya National Health Insurance Fund has started to enrol small, predominantly faith-based private facilities to provide free maternity services to all women who do not have health insurance coverage.^41^ As this programme is implemented, it will be critical for the Government of Kenya to develop strong systems for regulating the private sector and regularly monitoring the quality of care offered by participating providers.

Free maternity care in Kenya, like the voucher programme, is an output-based approach in which facilities are reimbursed per individual claim submitted for services provided. In many countries in sub-Saharan Africa, approaches that involve direct payments to facilities have been stymied by challenges that facilities have experienced in receiving timely, predictable and adequate reimbursements.^42^ Facilities in Kenya have similarly reported delayed or insufficient reimbursements for services provided, as well as being overwhelmed with patients as a result of free maternity services.^23–25 43 44^ Thus, if improvements in service use due to the provision and expansion of free maternity services are to be sustained at a high-quality in the long term, it is imperative that these operational challenges are resolved.

This study also highlights the importance of understanding access to care across the continuum of maternal health services rather than tracking progress towards access to each service individually. Although use of 4+ ANC, facility births and PNC has increased over time in Kenya, fewer than one in four births in both voucher and comparison counties received all three services at the recommended timings. Ensuring that women receive timely care across the entire continuum of maternal health services is critical to achieving further reductions in maternal mortality.

In order to comprehensively understand the impact of the voucher programme, free maternity services and other health financing approaches in Kenya, future research needs to look into the longer-term effects of these initiatives on quality and continuum of care, equity in access and financial burden to women and their households. This information will help to identify key strategies for ensuring sustained improvements in maternal and child health outcomes in Kenya and other similar contexts.
