# Supplementary figures and images for: Evaluating the impact of a maternal health voucher programme on service use before and after the introduction of free maternity services in Kenya: a quasi-experimental study
Source: BMJ Glob Health. 2018 May 2;3(2):e000726. doi: 10.1136/bmjgh-2018-000726 (PMC5935164; doi:10.1136/bmjgh-2018-000726)

## Appendix 1: Counties included in analysis

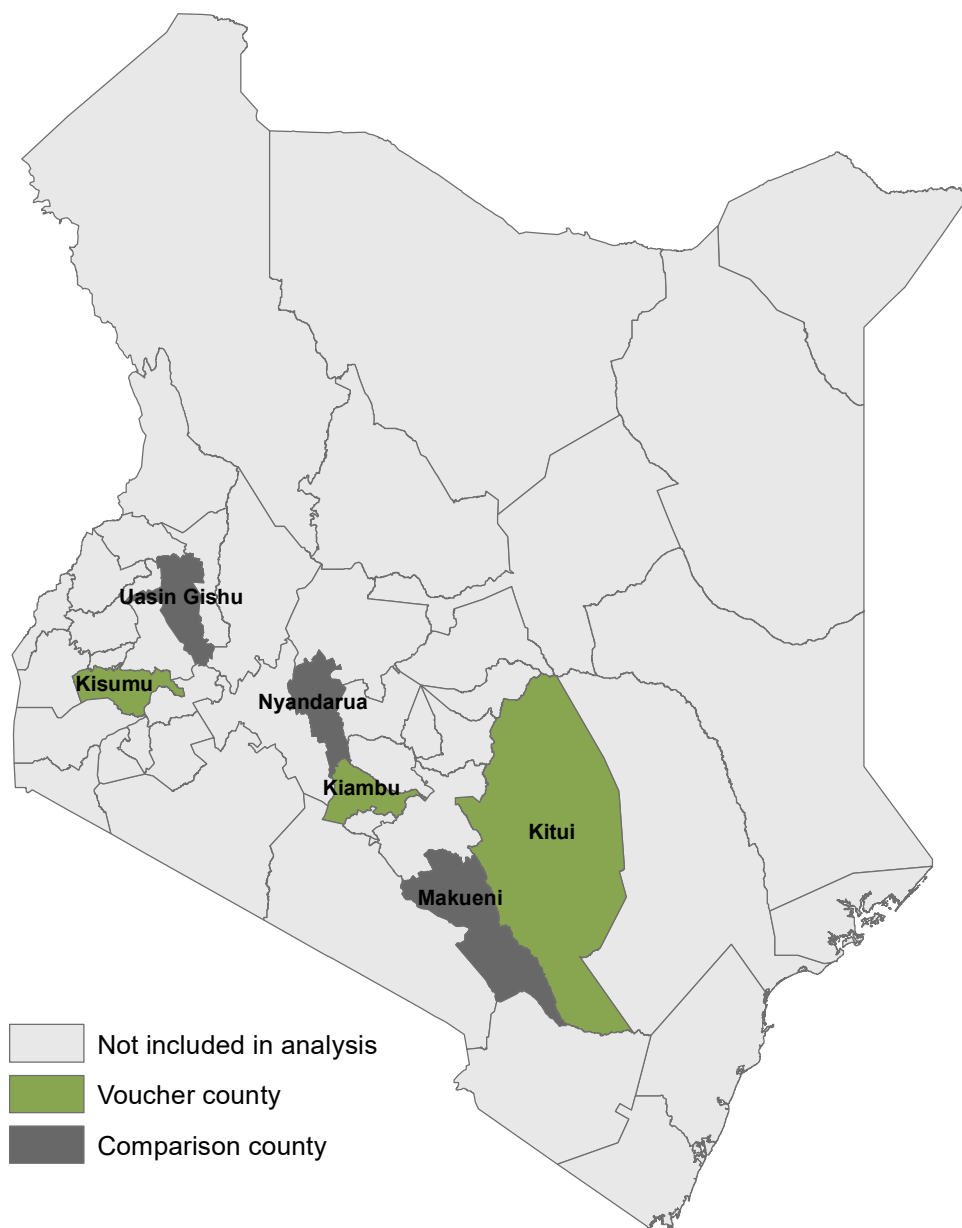

Supplement: Supplementary data [file bmjgh-2018-000726supp001.pdf]
