# Supplementary material for: Evaluating the impact of a maternal health voucher programme on service use before and after the introduction of free maternity services in Kenya: a quasi-experimental study
Source: BMJ Glob Health. 2018 May 2;3(2):e000726. doi: 10.1136/bmjgh-2018-000726 (PMC5935164; doi:10.1136/bmjgh-2018-000726)
Supplement: Supplementary data [file bmjgh-2018-000726supp002.pdf]

## Appendix 2: Description of missing data, 2016 dataset

In the 2016 dataset, 23% of women (N=621) who reported one or more births in their lifetime are missing data on the number of children they gave birth to in the past five years due to a glitch in the survey programming software (Figure A2.1). As the number of births in the past five years served as a filter question for the subsequent survey module on maternal health service utilization for each birth in the past five years, this glitch resulted in unit nonresponse, with women with missing information on this item lacking responses for all service utilization outcomes.

The glitch was identified during the course of fieldwork and corrected. As a result, the proportion of missing data declines towards the end of the survey, and relates to the respondent's county (Table A2.1). We examined the relationship between intervention group and likelihood of having missing data on the number of births in the past five years and did not find evidence of an effect in either the unadjusted or the adjusted models (Table A2.2). We also explored differences in the odds of having missing data by key sociodemographic factors. After adjusting for differences in background characteristics, we found that both marital status and county had strong effects on the odds of having missing data.

**Figure A2.1: Flow chart of number of participants with missing data, 2016 survey**

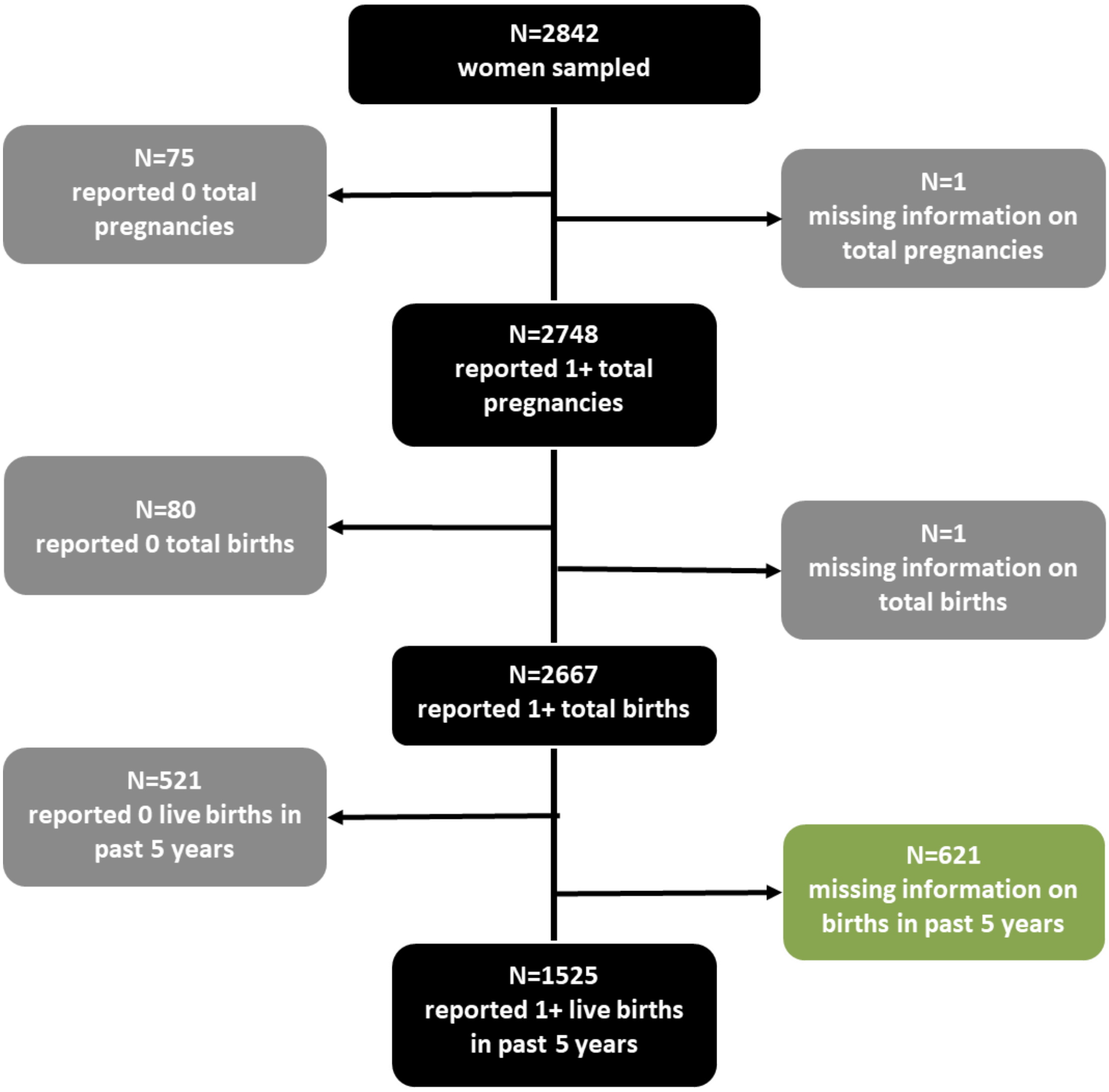

**Table A2.1: Missing data among women with 1+ total births by county, 2016 survey**

| | Survey dates | Proportion missing (%) |
|---|---|---|
| **County** | | |
| Kisumu (voucher) | 7 July 2016 – 23 July 2016 | 43.9 |
| Makueni (comparison) | 7 July 2016 – 23 July 2016 | 32.4 |
| Nyandarua (comparison) | 7 July 2016 – 23 July 2016 | 25.5 |
| Uasin Gishu(comparison) | 26 July 2016 – 12 Aug 2016 | 27.3 |
| Kiambu (voucher) | 26 July 2016 – 12 Aug 2016 | 5.4 |
| Kitui (voucher) | 26 July 2016 – 12 Aug 2016 | 8.4 |

**Table A2.2: Missing data among women with 1+ total births by intervention group, 2016 survey**

| | Comparison sites N=1385 | Voucher sites N=1282 | Unadjusted p-value | Adjusted* p-value |
|---|---|---|---|---|
| **Proportion of respondents:** | | | p=0.095 | p<0.001 |
| No missing data | 71.7 | 82.1 | | |
| With missing data | 28.3 | 17.9 | | |

*Logistic regression adjusted for age, education, wealth quintile, residence, marital status, employment, parity, and county & accounting for multi-stage sampling at county sub-location & village levels

**Table A2.3: Odds of having missing data by background characteristics, 2016 survey**

| | Unadjusted odds ratio | Unadjusted p-value | Adjusted odds ratio* | Adjusted p-value* |
|---|---|---|---|---|
| **Age group (years) (%)** | | | | |
| 15-24 (ref) | 1.00 | | 1.00 | |
| 25-34 | 0.80 | 0.095 | 1.05 | 0.702 |
| 35+ | 0.41 | <0.001 | 0.68 | 0.054 |
| **Educational attainment (%)** | | | | |
| None | 0.90 | 0.772 | 1.29 | 0.469 |
| Primary (ref) | 1.00 | | 1.00 | |
| Secondary & above | 1.56 | <0.001 | 1.28 | 0.055 |
| **Wealth quintile (%)** | | | | |
| Poorest (ref) | 1.00 | | 1.00 | |
| Poorer | 0.86 | 0.433 | 0.82 | 0.307 |
| Middle | 1.15 | 0.492 | 1.03 | 0.874 |
| Richer | 0.96 | 0.859 | 0.83 | 0.488 |
| Richest | 1.67 | 0.093 | 1.28 | 0.444 |
| **Residence** | | | | |
| Rural (ref) | 1.00 | | 1.00 | |
| Urban | 2.03 | 0.155 | 1.89 | 0.202 |
| **Current marital status (%)** | | | | |
| Unmarried (ref) | 1.00 | | 1.00 | |
| Married/cohabiting | 1.86 | <0.001 | 1.85 | <0.001 |
| **Woman's employment (%)** | | | | |
| Unemployed (ref) | 1.00 | | 1.00 | |
| Informally employed | 0.73 | 0.044 | 0.99 | 0.932 |
| Formally employed | 0.54 | 0.007 | 0.80 | 0.379 |
| **Parity (%)** | | | | |
| 1 (ref) | 1.00 | | 1.00 | |
| 2-3 | 0.83 | 0.087 | 0.83 | 0.214 |
| 4-6 | 0.64 | 0.001 | 0.80 | 0.256 |
| 7+ | 0.48 | 0.005 | 0.70 | 0.226 |
| **County (%)** | | | | |
| Kiambu (v) (ref) | 1.00 | | 1.00 | |
| Nyandarua (c) | 5.95 | 0.001 | 7.13 | 0.001 |
| Kisumu (v) | 13.64 | <0.001 | 12.95 | <0.001 |
| Uasin Gishu (c) | 6.54 | <0.001 | 6.43 | <0.001 |
| Kitui (v) | 1.61 | 0.162 | 1.76 | 0.178 |
| Makueni (c) | 8.36 | <0.001 | 8.73 | <0.001 |

*Logistic regression adjusted for all other variables in table & accounting for multi-stage sampling at county sub-location & village levels
